# Effect of Text Message Reminders and Vaccine Reservations on Adherence to a Health System COVID-19 Vaccination Policy: A Randomized Clinical Trial

**DOI:** 10.1001/jamanetworkopen.2022.22116

**Published:** 2022-07-20

**Authors:** Mitesh S. Patel, Richard Fogel, Angela L. Winegar, Charles Horseman, Allison Ottenbacher, Saleem Habash, Jonathan L. Dukes, Teresa C. Brinson, Shanda C. Price, Frederick A. Masoudi, Joseph Cacchione, Baligh R. Yehia

**Affiliations:** 1 Ascension, St Louis, Missouri

## Abstract

**Importance:**

Many organizations implemented COVID-19 vaccination requirements during the pandemic, but the best way to increase adherence to these policies is unknown.

**Objective:**

To evaluate if behavioral nudges delivered through text messages could accelerate adherence to a health system’s COVID-19 vaccination policy.

**Design, Setting, and Participants:**

This randomized clinical trial was conducted within Ascension health system from October 11 to November 8, 2021. Participants included health system employees in the Midwest or South US who were not adherent with the vaccination policy 1 month before its deadline. Data were analyzed from November 17, 2021, to February 25, 2022.

**Interventions:**

Participants were randomly assigned to control or to receive a text message intervention that stated a vaccine had been reserved for the participant, with a scheduled date for vaccination within a 2-week period. Participants could reschedule to a different date within the period or upload a copy of their vaccination card. Follow-up text message reminders were sent the day before and the day of the appointment.

**Main Outcomes and Measures:**

The primary outcome was adherence to the health system’s vaccination policy during the 2-week intervention. Secondary outcomes included time to vaccination during a 4-week follow-up period.

**Results:**

The sample included 2000 participants (mean [SD] age, 36.4 [12.3] years; 1724 [86.2%] women), with 1000 participants randomized to the control group and 1000 participants randomized to the intervention group. Overall, there were 164 Hispanic participants (8.2%), 46 non-Hispanic Asian participants (2.3%), 202 non-Hispanic Black participants (10.1%), and 1418 non-Hispanic White participants (70.9%). By the end of the 2-week intervention, 363 participants in the text message nudge group (36.3%) and 318 participants in the control group (31.8%) were adherent with the vaccination policy, representing a significant increase of 4.9 (95% CI, 0.8 to 9.1) percentage points in adjusted analyses comparing the nudge group with the control group (*P* = .02). Among participants who became adherent by the end of the 4-week follow-up period, the text message nudge significantly reduced time to adherence by a mean of 2.4 (95% CI, 2.1 to 4.7) days (*P* < .001) and a median of 5.0 (95% CI, 2.5 to 7.7) days (*P* < .001) compared with the control group. At 4 weeks, overall vaccination adherence was no longer different between groups (control: 477 participants [47.7%]; intervention: 472 participants [47.2%]).

**Conclusions and Relevance:**

This randomized clinical trial found that a behavioral nudge delivered through text messages accelerated adherence to a health system’s COVID-19 vaccination policy but did change overall adherence by the time of the policy deadline.

**Trial Registration:**

ClinicalTrials.gov Identifier: NCT05037201

## Key Points

**Question:**

Can a behavioral nudge delivered through text messages with a reserved date for vaccination over a 2-week period accelerate employee adherence with a health system COVID-19 vaccination policy?

**Findings:**

In this randomized clinical trial of 2000 participants, the behavioral nudge delivered through text messages significantly increased adherence to the health system COVID-19 vaccination policy by 4.9 percentage points compared with the control group during the 2-week intervention period. At the 4-week time point near the vaccination policy deadline, there was no longer a significant difference in the overall adherence rate between groups.

**Meaning:**

This randomized clinical trial found that a behavioral nudge delivered by text message with a reserved date for vaccination accelerated adherence to a health system COVID-19 vaccination policy; however, other approaches may be needed to change overall adherence rates by the time of the policy deadline.

## Teaser

This randomized clinical trial evaluates use of a text message behavioral nudge to accelerate adherence to a health system’s COVID-19 vaccination policy.

## Introduction

COVID-19 has caused significant morbidity and mortality in the United States and around the world.^1,2^ While several effective vaccines are available, many people in the US have not been vaccinated.^3^ To overcome vaccine hesitancy, large employers and government agencies have implemented a number of approaches, from educational campaigns to financial incentives.^4^ However, early evidence suggests that the changes associated with these interventions have been limited, particularly for individuals with greater vaccine hesitancy.^5,6,7^

More recently, policy makers and large employers have implemented mandates for employees to receive COVID-19 vaccination. On July 29, 2021, President Biden signed an Executive Order that federal employees had to become vaccinated or participate in weekly COVID-19 testing.^8^ The Veterans Health Administration (VHA) implemented a vaccination mandate in July 2021 without the option for weekly testing. VHA employees who did not obtain vaccination by the deadline were suspended.^9^ In November 2021, the Department of Labor’s Occupational Safety and Health Administration (OSHA) expanded this policy to organizations with 100 or more employees, and the Centers for Medicare & Medicaid Services (CMS) announced a new requirement that all health care workers at CMS-covered facilities be vaccinated.^10^ By the Fall of 2021, more than 150 health systems across the United States had announced new policy requirements for their employees to receive COVID-19 vaccination. While there is precedent for vaccine mandates,^11^ acceptance among the general public has varied.^7^

Early reports indicated that these vaccine mandates increased employee vaccination rates to greater than 90% at a number of organizations.^12,13,14^ While these efforts have been a tremendous success, several challenges remain. First, increasing vaccination rates may still take time as many mandates provide 4 or more months between their announcement and their deadlines. Second, organizations need to plan to replace staff who chose to leave instead of getting vaccinated, which can be difficult to predict until very close to the mandate deadline. From an employer perspective, vaccine adherence includes the employee both being vaccinated and submitting documentation, or otherwise having an approved vaccination exemption. These steps are often delayed. Therefore, there is a significant need for new and innovative approaches to accelerate adherence to a COVID-19 vaccine mandate.

Nudges are subtle changes to the way information is framed or how choices are offered that can have an outsized impact on behavior.^15^ Nudges have been demonstrated to increase other types of vaccination, such as influenza.^16^ A randomized clinical trial conducted at a university found that emailing participants a default time and location for vaccination (opt-out condition) led to a 36% relative increase in vaccination compared with individuals who were sent an email to schedule an appointment (opt-in condition).^17^ In another study, patients from 2 large health systems were sent text messages in the days preceding a visit with their primary care clinician.^18^ The trial randomly assigned patients to receive 1 of 19 different text messaging approaches or to be in a control group. On average, nudges sent by text message increased vaccination by 5% compared with the control group, and several of the top-performing messages used the phrase “the vaccine has been reserved for you,” with up to an 11% increase in vaccination. This phrase leverages the principle of loss aversion, which has found that individuals are more motivated to avoid losing something they feel is already theirs than to do an equivalent amount of work to gain a similar benefit. Nudges have also been used for COVID-19 vaccination efforts. In a randomized clinical trial conducted at UCLA Health, researchers found that telling patients a vaccine was available to them and they should claim it increased COVID-19 vaccination by 6% compared with a control group.^19^ However, this trial was implemented earlier in the pandemic, when demand for vaccination exceeded supply. Therefore, we lack good evidence on how to use nudges among individuals who may be more vaccine hesitant.

In this study, our objective was to conduct a randomized clinical trial to evaluate whether a behavioral nudge sent by text message could accelerate adherence to a health system’s policy requiring COVID-19 vaccination. The text message provided the participant with an appointment date for vaccination, with options to change the date or upload vaccination documentation if they had already been vaccinated. The intervention was conducted in the month before the vaccination policy deadline and targeted a more vaccine-hesitant group of employees.

## Methods

The protocol for this randomized clinical trial was approved by the Ascension St. Vincent Institutional Review Board and is available in Supplement 1. Informed consent was waived because this was a pragmatic evaluation of a health system initiative posing minimal risk to participants. No compensation was provided to participants in the study. The trial followed the Consolidated Standards of Reporting Trials (CONSORT) reporting guideline.

### Study Design

This was a 2-arm randomized clinical trial conducted among 2000 health system employees from the Midwest and South regions of the United States who were not adherent with a policy requiring COVID-19 vaccination announced by Ascension health system on July 27, 2021, with a deadline of November 12, 2021. Ascension is 1 of the largest nonprofit health systems in the United States and operates more than 140 hospitals. The trial was conducted in the final month before the deadline and included a 2-week prespecified intervention period (October 11-25, 2021), during which participants in the intervention were notified by text message of a default appointment date for COVID-19 vaccination. The prespecified secondary outcome followed adherence rates for an additional 2-week follow-up period until November 8, 2021. Prior to the intervention, all participants received communications about the vaccination requirement through multiple channels, including email, but not through text messages. Employees not adherent by the deadline would be placed on suspension.

### Sample

Eligible participants included health system employees who were identified as having not submitted documentation of vaccination by October 5, 2021, the time data were obtained for randomization. Participants were classified as physicians, physician support roles, nursing, nursing support roles, or other type of employee. Data on participant characteristics were obtained from human resources (HR) records, and data on vaccination adherence were obtained from Associate and Occupational Health records at Ascension’s health systems. Race and ethnicity were self-reported in HR records and categorized as Hispanic, non-Hispanic Asian, non-Hispanic Black, non-Hispanic White, and other (including non-Hispanic American Indian or Alaskan Native, non-Hispanic Pacific Islander, and participants with more than 1 race) or unknown.

### Randomization

From the available list of employees who were not adherent and with a documented phone number and email address on record, we randomly selected 2000 for participation in the trial. From this group, we randomly assigned half to the control group and the other half to the intervention group using an electronic randomization process. All investigators and statisticians were blinded to group assignment until the trial and analysis plan were completed.

### Interventions

Prior to the initiation of the trial, all health system employees were informed of the vaccination requirement and the potential for job suspension or termination for nonadherence by November 12, 2021. These communications began when the mandate was implemented on July 27, 2021, and were delivered mostly by email and the intranet, as well as verbal communication during meetings with their hiring manager or supervisor. These types of communications continued during the trial for both groups. The control group received no other interventions.

The intervention group received an initial text message on October 11, 2021, stating that as an employee of the health system, vaccination was required; that a vaccine has been reserved for them with a specific date during the upcoming 2 weeks; and indicating they could come in at any time during hours of operation. This was followed by a confirmation text message. Examples of the text messages are available in the eFigure in Supplement 2. The text message provided either the name and location of a vaccination clinic in their area or a link to review clinic locations and hours of operation. The text message provided a link to schedule at another date or location and a link to upload their vaccination card if they had already received it. Participants received a reminder text message the day before and day of their appointment. Since some employee phone numbers from the HR system may have been outdated or not linked to a cell phone, we also provided a confirmation via email that went out after the first text message, with the same links to reschedule or upload their vaccine card. Text messages were sent using 2 messaging platforms. The initial text message was sent by Genesys, and follow-up text messages and emails were sent using Radix Health.

### Outcome Measures

The primary outcome measure was adherence to the vaccine policy by the end of 2 weeks after the start of the intervention. This measure required that an employee received at least 1 dose of a vaccine and submitted documentation of vaccination. We selected 2 weeks because the default appointment was set for within 2 weeks of the initial text message. Secondary outcome measures were time to vaccine documentation submission (mean and median days) within 4 weeks of the start of the intervention.

### Statistical Analysis

A priori power calculations estimated that a sample of 1450 participants would provide at least 90% power to detect a 5–percentage point difference in the adherence rate between the intervention and control groups. This assumed a baseline adherence rate of 20% and used a 2-sided α = .05.

All randomly assigned participants were included in the intention-to-treat analysis. Unadjusted analyses tabulated the percentage of participants in each group who became adherent over time. To obtain the adjusted differences in adherence in percentage points along with 95% CIs, we fit a linear probability model using the participant as the unit of analysis.^20^ The model was adjusted for employee job role (ie, physician, physician support, nursing, nursing support, or other), which was the only participant characteristic that differed significantly between randomized study arms. A quantile regression model was used to estimate adjusted differences in median days to adherence and bootstrapping with 200 cycles was used to estimate 95% CIs.^21,22^

We conducted exploratory subgroup analyses by participant characteristics with *t* tests or χ^2^ tests, as appropriate. We also conducted a per-protocol analysis excluding participants who were adherent prior to intervention implementation or those granted a vaccine exemption by the policy deadline. We used 2-sided hypothesis tests with a significance level of *P* = .05. All analyses were performed in R statistical software version 4.0.5 (R Project for Statistical Computing). Data were analyzed from November 17, 2021, to February 25, 2022.

## Results

### Sample Characteristics

**Figure 1.**
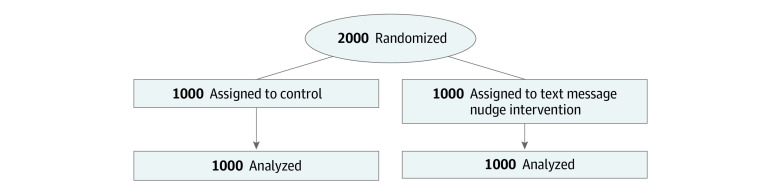
Participant Recruitment Flowchart

In this randomized clinical trial, 2000 health system employees were randomized, with 1000 participants in the intervention group and 1000 participants in the control group (Figure 1). Participants had a mean (SD) age of 36.4 (12.3) years and 5.2 (6.9) years at the health system; 1724 (86.2%) were women. The total sample included 164 Hispanic participants (8.2%), 46 non-Hispanic Asian participants (2.3%), 202 non-Hispanic Black participants (10.1%), and 1418 non-Hispanic White participants (70.9%) (Table 1). Employee job role was the only characteristic that was significantly different between the intervention and control groups and overall were represented in the sample as follows: 16 physicians (0.8%), 98 physician support employees (4.9%), 770 nurses (38.5%), 345 nursing support employees (17.3%), and 771 employees in other roles (38.6%).

**Table 1.** Sample Characteristics

Characteristic	No. (%)		
	Control (n = 1000)	Intervention (n = 1000)	Overall (N = 2000)
Age, mean (SD), y	36.0 (12.1)	36.7 (12.5)	36.4 (12.3)
Gender			
Men	149 (14.9)	127 (12.7)	276 (13.8)
Women	851 (85.1)	873 (87.3)	1724 (86.2)
Race and ethnicity			
Hispanic	88 (8.8)	76 (7.6)	164 (8.2)
Non-Hispanic			
Asian	15 (1.5)	31 (3.1)	46 (2.3)
Black	98 (9.8)	104 (10.4)	202 (10.1)
White	717 (71.7)	701 (70.1)	1418 (70.9)
Other^a^	72 (7.2)	80 (8.0)	152 (7.6)
Unknown	10 (1.0)	8 (0.8)	18 (0.9)
Insurance			
Health system employee plan	534 (53.4)	538 (53.8)	1072 (53.6)
Other	466 (46.6)	462 (46.2)	928 (46.4)
Role			
Physician	13 (1.3)	3 (0.3)	16 (0.8)
Physician support	40 (4.0)	58 (5.8)	98 (4.9)
Nursing	375 (37.5)	395 (39.5)	770 (38.5)
Nursing support	167 (16.7)	178 (17.8)	345 (17.3)
Other	405 (40.5)	366 (36.6)	771 (38.6)
Job type			
Full-time	702 (70.2)	653 (65.3)	1355 (67.8)
Part-time	95 (9.5)	118 (11.8)	213 (10.7)
Other	203 (20.3)	229 (22.9)	432 (21.6)
Health system employment, y			
<1 y	301 (30.1)	267 (26.7)	568 (28.4)
1 to <5	330 (33)	374 (37.4)	704 (35.2)
≥5	369 (36.9)	359 (35.9)	728 (36.4)

^a^ Other race and ethnicity includes non-Hispanic American Indian or Alaskan Native, non-Hispanic Pacific Islander, and participants with more than 1 race.

### Vaccination Adherence

When the intervention began, the overall vaccination adherence rate at the health system was 83.7%. Table 2 displays the vaccination adherence outcomes by study group and over time. Among all participants, 229 (11.5%) became adherent after randomization but before the intervention was implemented. In the intervention group 251 participants (25.1%) became adherent during the intervention period, compared with 201 participants (20.1%) in the control arm (adjusted difference, 5.6 [95% CI, 1.8 to 9.2] percentage points; *P* = .003). In the adjusted intention-to-treat analysis during the 2-week intervention period, 363 intervention participants (36.3%) were adherent by the end of the period, compared with 318 control participants (31.8%), a significant absolute increase of 4.9 (95% CI, 0.8 to 9.1) percentage points in vaccination adherence in the text message nudge group compared with the control group (*P* = .02), representing a 15.4% relative increase. No adverse events were reported during the trial.

**Table 2.** Vaccination Adherence Outcomes^a^

Outcome measure	No. (%)		Adjusted difference (95% CI), percentage points	*P* value
	Control (n = 1000)	Intervention (n = 1000)		
Adherent by end of the primary intervention period (2 wk)	318 (31.8)	363 (36.3)	4.9 (0.8 to 9.1)	.02
Became adherent before period began	117 (11.7)	112 (11.2)	−0.6 (−3.4 to 2.2)	.69
Became adherent during period	201 (20.1)	251 (25.1)	5.6 (1.8 to 9.2)	.003
Time to adherence during follow-up (4 wk), d				
Mean (SD)	10.3 (9.6)	7.87 (9.1)	−2.4 (−4.7 to −2.1)	<.001
Median (IQR)	9.0 (0.0 to 20.0)	3.0 (0.0 to 13.3)	−5.0 (−7.7 to −2.5)	<.001

^a^ Models are adjusted for employee job role, which was the only significantly different participant characteristic between the control and intervention groups.

Results were similar in the per-protocol analysis excluding the 404 participants (20.2% of the sample) who either became adherent before the intervention was implemented or had a vaccine exemption approved by the policy deadline, with a 6.0 (95% CI, 1.6 to 10.3) percentage point increase in the text message nudge group compared with the control group (*P* = .007) (eTable 1 in Supplement 2). Exploratory subgroup analyses indicated a larger magnitude difference among participants aged 36 to 50 years (25.8% adherence in the control group vs 34.9% adherence in the intervention group) (eTable 2 in Supplement 2).

### Time to Vaccination Adherence

**Figure 2.**
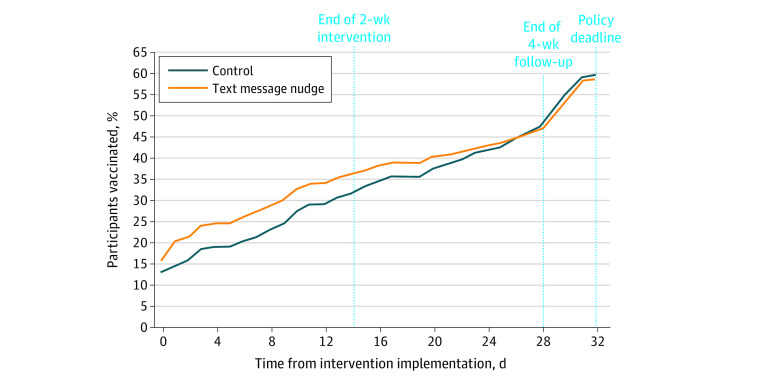
Vaccination Adherence by Time

Among participants who became adherent by the end of the 4-week follow-up period, the text message nudge significantly reduced time to adherence by a mean of 2.4 (95% CI, 2.1 to 4.7) days (*P* < .001) and a median of 5.0 (95% CI, 2.5 to 7.7) days (*P* < .001) compared with the control group (Table 2). The percentage of participants who became adherent over time from trial implementation until the policy deadline is presented in Figure 2. Adherence rates became similar in the final week before the policy deadline (control: 477 participants [47.7%]; intervention: 472 participants [47.2%]).

## Discussion

In this randomized clinical trial, a nudge delivered by text message stating the COVID-19 vaccine had been reserved for them on a specific appointment date significantly increased adherence to the health system vaccination policy during the 2-week intervention. The nudge also significantly accelerated the time to adherence within 4 weeks. These findings demonstrate the potential impact of using nudges to enhance timely adherence to a vaccine requirement. However, overall adherence did not differ by the time of the vaccination policy deadline, indicating that other approaches may be needed.

Our findings have several important implications for policy makers and large employers who have implemented or are considering a mandate for COVID-19 vaccination. First, our work demonstrates that behavioral nudges can be effective for accelerating vaccination adherence among a more vaccine-hesitant population. The COVID-19 vaccine was first made available to health care workers in late 2020; therefore, nearly a year had passed and the participants of this randomized clinical trial had not been vaccinated. Our work builds on previous nudge interventions that framed influenza vaccination as opt-out and told participants the vaccine was reserved for them.^17,18^ However, it differs from a UCLA Health study that used text message nudges for COVID-19 much earlier in the pandemic among a larger group of individuals who had not been vaccinated and who were likely not as vaccine hesitant.^19^ Importantly, the 15.4% relative increase in vaccination adherence within this trial was larger in magnitude than the 5% relative increase in influenza vaccination found in the study by Milkman et al^18^ and the 6% relative increase in COVID-19 vaccination found in the study by Dai et al,^19^ each of which were likely implemented in less vaccine-hesitant populations than in our study.

Second, the behavioral nudges implemented in this trial were particularly important for accelerating the time to adherence. For large employers, faster adherence can offer both a safer environment for the workforce and better management of future staffing needs. An employee can face delays in both obtaining vaccination and submitting documentation. The intervention prompted action by providing a prespecified date for vaccination but also enabled employees to reschedule if another date worked better for them. Once vaccinated, participants could use the link in the text message to upload a picture of their vaccination card. Since most previous communications were by email or verbally, this text message approach may have offered enhanced salience. These vaccination reservations may have also simply made it easier for participants to follow through with vaccination, which indicates that health systems should consider looking for ways to improve structural changes to the delivery of health care services.

Third, in the final week before the vaccination policy deadline, approximately 15% of the sample became adherent, and rates between groups became similar. Similar to prior reports,^12,13,14^ this demonstrates the strong association of these types of policies with increasing vaccination. However, it also demonstrates that many employees will put off vaccination unless prompted earlier, highlighting the importance of using behavioral nudges to improve the speed of adherence. These findings also indicate that other approaches may be needed to change overall adherence rates when interventions are implemented close to the policy deadline. Future research could evaluate these types of approaches when implemented closer to the mandate announcement than its deadline.

Fourth, the ongoing COVID-19 pandemic and new strains of the virus indicate that regular vaccination may be needed for some period. In November 2021, the Centers for Disease Control and Prevention recommended that all adults receive a booster vaccination at least 3 months after completing the initial series.^23^ Adherence to booster vaccination has been low.^24^ Behavioral nudges could also provide an important approach to address this issue.

### Limitations

Our study has limitations. First, it was conducted among employees from within the same health system, which could limit the generalizability of the results to other populations. Second, we had a shorter follow-up period, given that the trial was conducted in the last month before a COVID-19 vaccination requirement deadline. Third, we focused on adherence to the health system vaccination policy, which includes both obtaining vaccination and submitting documentation. Participants obtaining vaccination outside of Ascension facilities could have delayed submitting documentation. However, from an employer perspective, adherence relied on documentation submission. We did not have data on participants who received vaccination but did not submit documentation of it. Fourth, adherence was prespecified as obtaining the first COVID-19 vaccination dose, and some vaccines require a second dose to complete the series. This was designed to be aligned with health system adherence policies. Fifth, we were unable to quantify how many participants had phone numbers that were outdated or a landline, thereby preventing them from receiving the text message nudge. However, this represents a pragmatic implementation of the approach and would bias our results toward the null.

## Conclusions

The findings of this randomized clinical trial suggest that behavioral nudges delivered through text messages offered a scalable approach to accelerate adherence and could be adopted more broadly to improve COVID-19 vaccination efforts. However, other approaches may be needed for interventions implemented close to the policy deadline to impact overall adherence rates.

## Notes

Supplement 1.
Trial Protocol and Statistical Analysis Plan

Supplement 2. **eFigure.** Text Message Nudge Intervention **eTable 1.** Vaccination Adherence in Per Protocol Analysis **eTable 2.** Vaccination Adherence Outcomes by Participant Subgroups

Supplement 3.
Data Sharing Statement
