# Supplementary material for: Effect of Text Message Reminders and Vaccine Reservations on Adherence to a Health System COVID-19 Vaccination Policy: A Randomized Clinical Trial
Source: JAMA Netw Open. 2022 Jul 20;5(7):e2222116. doi: 10.1001/jamanetworkopen.2022.22116 (PMC9301516; doi:10.1001/jamanetworkopen.2022.22116)

A Randomized Trial of Text Message-Based Nudges to Increase COVID-19 Vaccination

August 27, 2021

1. Abstract

The COVID-19 pandemic has caused significant morbidity and mortality across the world. Effective vaccines are now available but underutilized. In July 2021, Ascension Health implemented a mandate requiring all employees to obtain the COVID-19 vaccine by November 12th. In August 2021, the number of COVID-19 cases in the US increased rapidly, specifically in states with lower vaccination rates, many of which are served by Ascension Health facilities. In this study, we will evaluate a rapidly deployed health system initiative to use text messaging to nudge Ascension employees who have not yet been vaccinated to commit to a date and receive vaccination.

2. Overall objectives

To evaluate if using text messages to set a default date for COVID-19 vaccination can encourage higher and faster vaccination rates.

3. Aims

3.1 Primary outcome

The percent of participants that receive the COVID-19 vaccine within 2 weeks of the intervention.

3.2 Secondary outcome

Time to COVID-19 vaccination in days within 4 weeks of the intervention.

4. Background

The COVID-19 pandemic has caused significant morbidity and mortality across the world. Effective vaccines are now available but underutilized. In July 2021, Ascension Health implemented a mandate requiring all employees to obtain the COVID-19 vaccine by November 12th. In August 2021, the number of COVID-19 cases in the US increased rapidly, specifically in states with lower vaccination rates, many of which are served by Ascension Health facilities.

Nudges are subtle changes to the way information is framed or choices are offered that can have a significant impact on behavior. Status quo bias is a preference for the current state which is often used as a reference point when making decisions. Changes from the reference point are often faced with high inertia and this prevents changes in behavior. Default options are the setting of the baseline reference point and are often taken as an implicit recommendation.

In prior work, changing default options has led to significant changes in health-related behaviors. For example, changing prescription default settings in the EHR from opt-in to opt-out for generic prescriptions increased the rate of generic prescriptions from 75% to 98% across Penn Medicine (Patel et al. Annals of IM. 2014; Patel et al. JGIM 2018). Framing program participation as opt-out has led to a 22% increase in enrollment in a COVID-19 surveillance testing program (Oakes et al. NEJM Catalyst 2021) and triple enrollment in remote monitoring programs for medication adherence (Mehta et al. JAMA Cardiology. 2018) and diabetes management (Aysola et al. AJHP 2016)

5. Study Design

5.1 Design

This will be conducted as a two-arm randomized, controlled trial. The control arm will receive usual health system messaging about the importance and deadlines for receiving COVID-19 vaccination. The intervention arm will receive a text message stating that the vaccine is reserved for them on a specific date. They will have the ability to reschedule to a different day, opt-out of this text messaging intervention, or if previously vaccinated they can upload documentation to the Ascension website.

A list of Associates for whom Ascension does not have documentation of vaccination or exemption from vaccination will be provided by Human Resources at Ascension. Information on vaccine clinic locations and hours are available online and a link to them will be provided in the text message.

Participants sites will likely include Ascension Associates from the following markets: DC, Maryland, NY Binghamton, Kansas and/or Florida

The trial is anticipated to begin around: September 13, 2021.

5.2 Study duration

This rapid health system intervention will be completed within 1 month. We will follow participant vaccination status from the time the interventions were sent out until 2 weeks after the end of the first week of vaccination dates.

5.3 Target population

Ascension Associates for whom the health system does not have documentation of COVID-19 vaccine or exemption from vaccination

5.4 Accrual

The sample will be identified by the health system and there will not be active participant recruitment.

5.5 Key inclusion criteria

To be eligible, participants must be age 18 or older and an Ascension Associate without documentation of vaccination or exemption at the time of the intervention implementation

6. Participant recruitment

The sample will be identified by the health system and there will not be active participant recruitment.

7. Participant compensation

There will be no compensation to participants in this study.

8. Study procedures

8.1 Consent

A waiver of informed consent is requested for the following reasons. First, this is a rapid deployment of a health system initiative and it will not be feasible to consent each participant in a timely manner. Second, the health system has already implemented a mandate for COVID-19 vaccination for the target population. Third, if the control group was consented, it could change their behavior differently from what it would be under usual care and that could hinder evaluation of the effectiveness of the intervention. Fourth, we provide the opportunity for all intervention participants to opt-out of messaging if they would like to do that.

8.2 Procedures

Prior to the interventions, all Associates identified in the selected markets will be sent an email from Associate Health informing them of the upcoming text message intervention, that it will be staggered so some get it before others, and which phone number it will be sent from. Participants in the intervention arm will be sent up the following text messages (See Section 12.1 for full text message script).. Message 1 will inform them of Ascension's goal of keeping everyone safe during the pandemic, that a vaccine is reserved for them on a specific day, URL with vaccine clinic locations and times, and ability to either reschedule to another day, or obtain a link to upload documentation of vaccination. Message 2 will be sent the day before the scheduled date with vaccine clinic location and hours as well as allow them to reschedule, or

upload documentation if needed. Message 3 will be sent the morning of the date scheduled with vaccine clinic location and hours as well as allow them to reschedule, or upload documentation if needed. Message 4 will be sent the day after the date scheduled to ask if the vaccine was received or not. If yes, then the link for uploading documentation will be sent. If not they will be scheduled for another day and cycle back to Message 1. If the approach is found to be successful, participants in control will receive the same intervention about 2 weeks later.

9. Analysis plan

Prior to analyses, we will produce data summaries to assess data quality, data distribution, and randomization success. All analyses will be evaluated using an intention-to-treat approach. An individual will be classified as vaccinated if they uploaded documentation of vaccination within 2 weeks after the end of the first week of vaccination appointments.

We estimate that a sample of 1450 (725 per arm) will provide at least 90% power to detect a 5 percentage point difference in vaccination between the intervention and control groups. This assumes a 20% vaccination rate in the control group and uses a two-sided alpha of 0.05.

The primary analysis will use a chi-square test to evaluate for differences in vaccination rate between the intervention and control group. Differences in time to vaccination will be evaluated using a t-test for means and Mann-Whitney test for medians. We will test the robustness of these findings by fitting a fully adjusted model with available participant characteristics. We will perform exploratory subgroup analyses for available participant characteristics.

10. Investigators

Mitesh Patel, MD, MBA is Vice President for Clinical Transformation and National Lead for Behavioral Insights, Clinical and Network Services at Ascension Health. In his previous role at Penn Medicine, he was an endowed, tenured Associate Professor and had led 25+ randomized clinical trials testing behavioral interventions to change clinician and patient behaviors.

11. Human research protection

11.1 Data confidentiality

Computer-based files will only be made available to study personnel through use of access privileges and passwords. Wherever possible, identifiers will be removed from study related information and analyses. Precautions will be in place for data security and HIPPA compliance.

11.2 Participant confidentiality

Information on participants will be obtained from the health system and will not be shared in a deidentified manner outside of the study team.The Ascension Data Science Institute (ADSI) will be the hub for data collection and evaluation. ADSI provides a secure platform with firewalls for management and evaluation of health system data including meeting standards for Health Insurance Portability and Account Act (HIPPA).

11.3 Participant privacy

All efforts will be made by study staff to ensure participant privacy. Data will be evaluated in a deidentified manner whenever possible.

11.4 Data disclosure

Information on patients will only be disclosed within the study team.

11.5 Data and safety monitoring

The Principal Investigator will closely monitor the safety, privacy and data integrity of the study.

11.6 Risk/benefit

11.6.1 Potential study risks

The main risk is data breach and as previously described precautions will be taken to minimize this risk

11.6.2 Potential study benefits

Successful approaches to encourage vaccination among more hesitant individuals could be important for other vaccination efforts to help address the COVID-19 pandemic

11.6.3 Risk/benefit assessment

There is minimal risk of breach of data and appropriate measures to reduce this risk have been taken. Given the potential benefits of the study, we believe the risk/benefit assessment is favorable.

12. Appendix

12.1 Text messaging script

Initial Text at 9am market time

[First name Last name], Ascension is committed to providing a safe environment for associates, patients and visitors. All associates are required to receive the COVID-19 vaccine series by November 12, 2021. A dose is reserved for you on [insert day of the week or date, e.g. Monday, August 30, 2021].

Vaccine clinic locations and hours can be found here: [insert Market specific URL]

Reply Reschedule if you need to select a different date. Reply Upload if already vaccinated and you need to upload your vaccine card.

- If Reschedule: What day works best for you? Reply Monday, Tuesday, Wednesday, Thursday, or Friday.
    - You are confirmed for [insert day selected and reset appointment date in system]
    - If Other: Sorry, we did not recognize that response. You can text back Monday, Tuesday, Wednesday, Thursday, or Friday.
- If Upload: Submit your vaccination verification here; you will need your employee ID number and a photo of your vaccine documentation: https://gdaintranet.ascension.org/home/covid-19-vaccine-external-documentation [then stop text messages]
- If Other: Sorry, we did not recognize that response. You can text back Reschedule or Upload. If that day works for you, then you can visit any clinic within the hours they are open on that day. You may also receive vaccination from another site and Upload your vaccine care. If questions, email: associaterelationscommunications@ascension.org

Example Screenshot

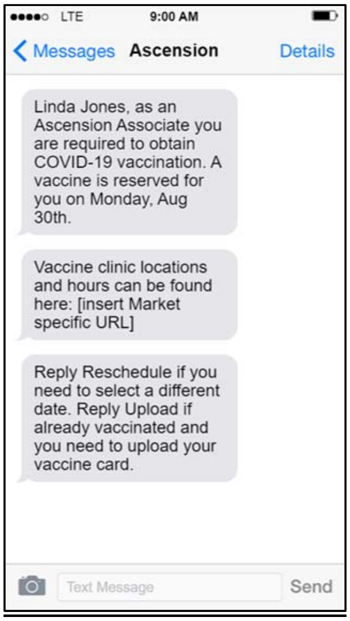

One day before scheduled date at 9am market time

A COVID-19 vaccine is reserved for you tomorrow. Locations and hours can be found here: [insert Market specific URL]

Reply Reschedule if you need to select a different day next week. Reply Upload if already vaccinated; you need to upload your vaccine card.

- If Reschedule: What day works best for you? Reply Monday, Tuesday, Wednesday, Thursday, or Friday.
  - You are confirmed for [insert day selected and reset appointment date in system]
  - If Other: Sorry, we did not recognize that response. You can text back Monday, Tuesday, Wednesday, Thursday, or Friday.
- If Upload: Submit vaccination verification here; you will need your employee ID number and a photo of your vaccine documentation: https://gdaintranet.ascension.org/home/covid-19-vaccine-external-documentation [then stop text messages]
- If Other: Sorry, we did not recognize that response. You can text back Reschedule or Upload. If that day works for you, then you can visit any clinic within the hours they are open on that day. You may also receive vaccination from another site and Upload your vaccine care. If questions, email: associaterelationscommunications@ascension.org

Example Screenshot

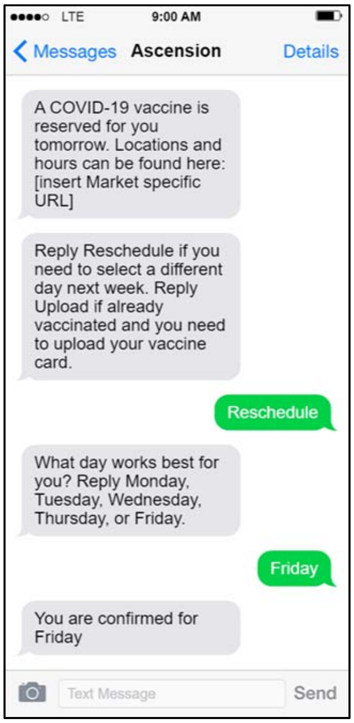

Morning of Day scheduled at 9am market time

A COVID-19 vaccine is reserved for you today. Locations and hours can be found here: [insert Market specific URL]

Reply Reschedule if you need to select a different day next week. Reply Upload if already vaccinated and you need to upload your vaccine card.

- If Reschedule: What day works best for you? Reply Monday, Tuesday, Wednesday, Thursday, or Friday.
    - You are confirmed for [insert day selected and reset appointment date in system]
    - If Other: Sorry, we did not recognize that response. You can text back Monday, Tuesday, Wednesday, Thursday, or Friday.
- If Upload: Submit vaccination verification here; you will need your employee ID number and a photo of your vaccine documentation: https://gdaintranet.ascension.org/home/covid-19-vaccine-external-documentation [then stop text messages]
- If Other: Sorry, we did not recognize that response. You can text back Reschedule or Upload. If that day works for you, then you can visit any clinic within the hours they are open on that day. You may also receive vaccination from another site and Upload your vaccine care. If questions, email: associaterelationscommunications@ascension.org

Example Screenshot

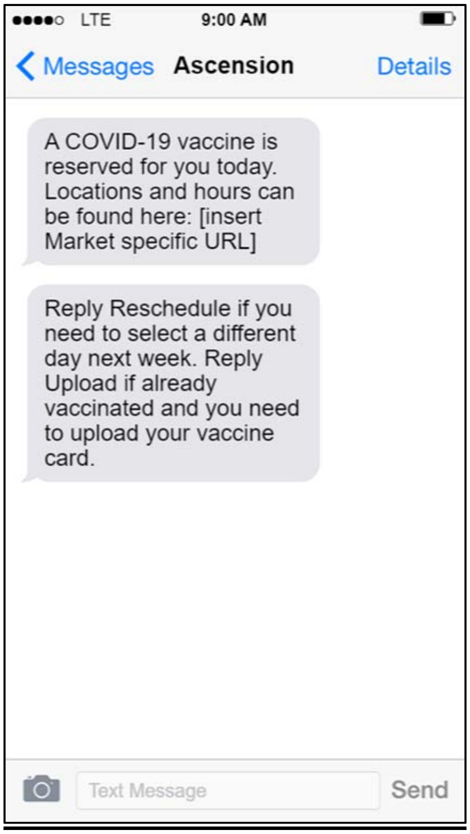

Day after appointment

Did you receive the COVID-19 vaccine yesterday? Reply Yes or No

- If Yes: Thank you for doing your part to create a safe environment for our associates, patients and visitors. If you got the vaccine at a site other than Associate Health, please upload an image of your vaccine card here: https://gdaintranet.ascension.org/home/covid-19-vaccine-external-documentation [then stop text messages]
- If No: A COVID-19 vaccine is reserved for you next Monday. Locations and hours can be found here: [insert Market specific URL]
    - Reply Reschedule if you need to select a different day next week. Reply Upload if already vaccinated and you need to upload your vaccine card. [restart from top of logic]
- If Other: Sorry, we did not recognize that response. You can text back Yes or No. If questions, email: associaterelationscommunications@ascension.org

Example Screenshot

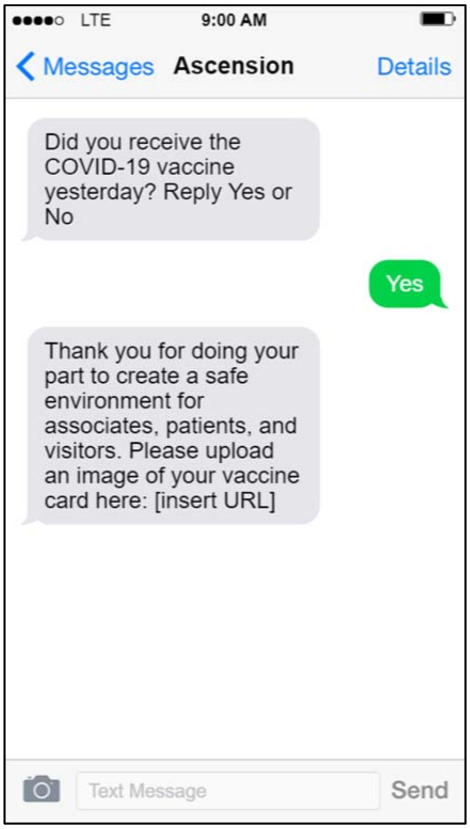

Supplement: Supplement 1. — Trial Protocol and Statistical Analysis Plan [file jamanetwopen-e2222116-s001.pdf]
