# Supplementary material for: Effect of Text Message Reminders and Vaccine Reservations on Adherence to a Health System COVID-19 Vaccination Policy: A Randomized Clinical Trial
Source: JAMA Netw Open. 2022 Jul 20;5(7):e2222116. doi: 10.1001/jamanetworkopen.2022.22116 (PMC9301516; doi:10.1001/jamanetworkopen.2022.22116)
Supplement: Supplement 2. — eFigure. Text Message Nudge Intervention eTable 1. Vaccination Adherence in Per Protocol Analysis eTable 2. Vaccination Adherence Outcomes by Participant Subgroups [file jamanetwopen-e2222116-s002.pdf]

Supplemental Online Content

Patel MS, Fogel R, Winegar AL, et al. Effect of text message reminders and vaccine reservations on adherence to a health system COVID-19 vaccination policy: a randomized clinical trial. *JAMA Netw Open*. 2022;5(7):e2222116. doi:10.1001/jamanetworkopen.2022.22116

eFigure. Text Message Nudge Intervention

eTable 1. Vaccination Adherence in Per Protocol Analysis

eTable 2. Vaccination Adherence Outcomes by Participant Subgroups

This supplemental material has been provided by the authors to give readers additional information about their work.

© 2022 Patel MS et al. *JAMA Network Open.*

eFigure. Text Message Nudge Intervention

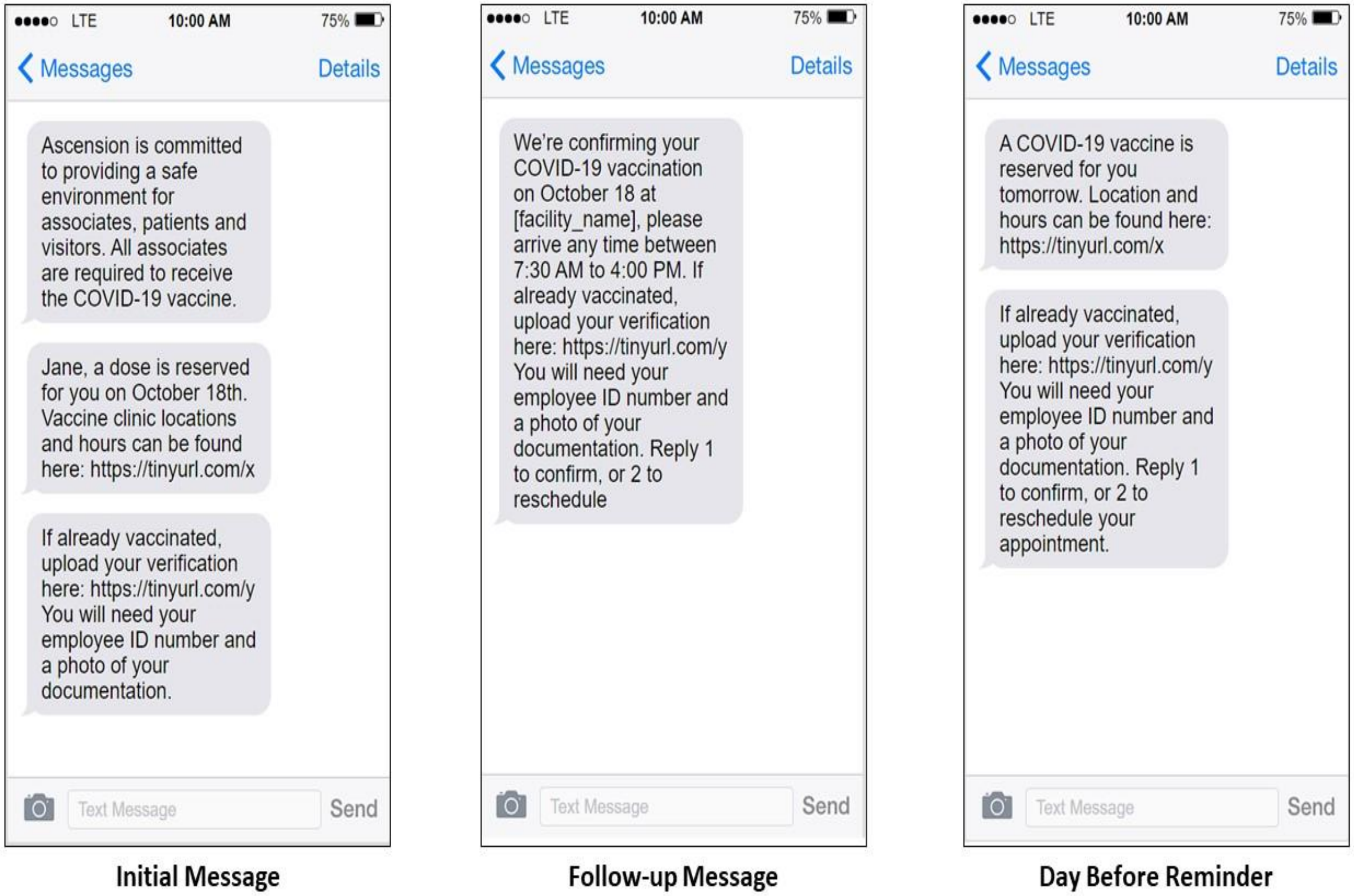

*An initial text message was sent (left) followed by a confirmation text message and email (middle). Participants were also sent a reminder on the day before the appointment (right). If a participant did not confirm the day before reminder, they were also sent a similar message on the day of the appointment.

© 2022 Patel MS et al. *JAMA Network Open.*

eTable 1. Vaccination Compliance in Per Protocol Analysis

Outcome Measure (Per Protocol Analysis)	Control	Intervention	Adjusted Difference and 95% CI (Intervention relative to Control)	P Value
Participants, No.	792	804	NA	NA
**Primary intervention period (2 weeks)**				
Compliant by end of the period, No. (%)	193 (24.37)	240 (29.85)	6.0 (1.6, 10.3)	**<0.01**
**Follow-up period (4 weeks)**				
Days until compliant, Mean (SD)	13.6 (8.7)	10.2 (9.1)	-3.4 (-4.8, -2.1)	**<0.001**
Days until compliant, Median (IQR)	13.0 (7.0, 21.0)	8.5 (1.0, 16.0)	-3.0 (-4.3, 0.4	**0.01**

*Analysis after removing participants who became compliant before the intervention was implemented or had a vaccine exemption request approved. Models are adjusted for employee job role which was the only significantly different participant characteristic between the control and intervention arms.

© 2022 Patel MS et al. *JAMA Network Open.*

eTable 2. Vaccination Compliance Outcomes by Participant Subgroups

**Characteristic**	**Control**	**Intervention**	**P Value**
Age			
18-35 years	203/558 (36.4)	212/541 (39.2)	0.37
36-50 years	77/299 (25.8)	102/292 (34.9)	**0.02**
51-64 years	35/123 (28.5)	40/143 (28.0)	1.00
65 years or older	3/20 (15.0)	9/24 (37.5)	0.18
Gender			
Female	259/851 (30.4)	306/873 (35.1)	**0.047**
Male	59/149 (39.6)	57/127 (44.9)	0.45
Race/ethnicity			
White non-Hispanic	214/717 (29.9)	232/701 (33.1)	0.21
Black non-Hispanic	37/98 (37.8)	41/104 (39.4)	0.92
Asian non-Hispanic	6/15 (40.0)	20/31 (64.5)	0.21
Hispanic	35/88 (39.8)	31/76 (40.8)	1.00
Other/Unknown	26/82 (31.7)	39/88 (44.3)	0.13
Insurance			
Health system employee plan	103/261 (39.5)	101/259 (39.0)	0.99
Health system employee plan with dependents	96/273 (35.2)	99/279 (35.5)	1.00
Other but eligible for employee plan	62/242 (25.6)	67/210 (31.9)	0.17
Other but Ineligible for employee plan	57/224 (25.5)	96/252 (38.1)	**0.004**
Role			
Physician	6/13 (46.2)	1/3 (33.3)	1.00
Physician support	7/40 (17.5)	22/58 (37.9)	0.05
Nursing	101/375 (26.9)	124/395 (31.4)	0.20
Nursing support	57/167 (34.1)	71/178 (39.9)	0.32
Other	147/405 (36.3)	145/366 (39.6)	0.38
Job Type			
Full-time	241/702 (34.3)	247/653 (37.8)	0.20
Part-time	26/95 (27.4)	31/118 (26.3)	0.98
Other	51/203 (25.1)	85/229 (37.1)	**0.01**
Health system employment			
Less than 1 year	115/301 (38.2)	116/267 (43.5)	0.24
1 to 5 years	105/330 (31.8)	141/374 (37.7)	0.12
5 years or more	98/369 (26.6)	106/359 (29.5)	0.42

*P Values compared the control and intervention groups across each row in the table.

© 2022 Patel MS et al. *JAMA Network Open.*
