# Supplementary material for: Effect of Text Message Reminders and Vaccine Reservations on Adherence to a Health System COVID-19 Vaccination Policy: A Randomized Clinical Trial
Source: JAMA Netw Open. 2022 Jul 20;5(7):e2222116. doi: 10.1001/jamanetworkopen.2022.22116 (PMC9301516; doi:10.1001/jamanetworkopen.2022.22116)
Supplement: Supplement 3. — Data Sharing Statement [file jamanetwopen-e2222116-s003.pdf]

Data Sharing Statement

Patel. Effect of Text Message Reminders and Vaccine Reservations on Adherence to a Health System COVID-19 Vaccination Policy. *JAMA Netw Open*. Published July 20, 2022. doi:10.1001/jamanetworkopen.2022.22116

Data
Data available: No
